# Behavioral recovery and spinal motoneuron remodeling after polyethylene glycol fusion repair of singly cut and ablated sciatic nerves

**DOI:** 10.1371/journal.pone.0223443

**Published:** 2019-10-04

**Authors:** Cameron L. Ghergherehchi, Emily A. Hibbard, Michelle Mikesh, George D. Bittner, Dale R. Sengelaub

**Affiliations:** 1 Department of Molecular Biosciences, University of Texas at Austin, Austin, Texas, United States of America; 2 Department of Psychological and Brain Sciences, Indiana University, Bloomington, Indiana, United States of America; 3 Department of Neuroscience, University of Texas at Austin, Austin, Texas, United States of America; University of Sydney, AUSTRALIA

## Abstract

Polyethylene glycol repair (PEG-fusion) of severed sciatic axons restores their axoplasmic and membrane continuity, prevents Wallerian degeneration, maintains muscle fiber innervation, and greatly improves recovery of voluntary behaviors. We examined alterations in spinal connectivity and motoneuron dendritic morphology as one potential mechanism for improved behavioral function after PEG-fusion. At 2–112 days after a single-cut or allograft PEG-fusion repair of transected or ablated sciatic nerves, the number, size, location, and morphology of motoneurons projecting to the tibialis anterior muscle were assessed by retrograde labeling. For both lesion types, labeled motoneurons were found in the appropriate original spinal segment, but also in inappropriate segments, indicating mis-pairings of proximal-distal segments of PEG-fused motor axons. Although the number and somal size of motoneurons was unaffected, dendritic distributions were altered, indicating that PEG-fusion preserves spinal motoneurons but reorganizes their connectivity. This spinal reorganization may contribute to the remarkable behavioral recovery seen after PEG-fusion repair.

## Introduction

Peripheral nerve injuries (PNIs) that disrupt axonal continuity (axotomy) have many effects on peripheral nervous system (PNS) structure and function, such as the immediate loss of voluntary motor control, sensory reception, rapid degeneration of the distal axonal segments (Wallerian degeneration), loss of neuromuscular junction (NMJ) innervation, and muscle fiber atrophy [1]. In addition to these PNS changes after PNIs, changes occur in the central nervous system (CNS) as well. For example, motoneurons in the spinal cord undergo alterations in response to axotomy. Although most motoneurons survive PNIs in adult mammals [2–4], motoneuron somata show many structural, functional and biochemical changes [5–7]. These PNI-induced changes include somal atrophy [4, 8] and withdrawal of synaptic inputs from somata and proximal dendrites [8–10]. Muscle reinnervation reverses the loss of some of these synaptic inputs [9], but other inputs are permanently lost, particularly IA afferent neurons that innervate muscle spindles [9, 10].

Axotomy of motoneurons also alters their dendritic morphology. For example, after distal sciatic PNIs, injured motoneurons have thicker proximal dendrites that sometimes abruptly taper [11] and retract [8, 12]. Permanent axotomy of gastrocnemius motoneurons reduces dendritic diameter within 3 weeks, and dramatically reduces dendritic membrane area and volume within 12 weeks [13]. Dendritic regressions after axotomy can be reversed upon muscle reinnervation [12, 14, 15]. Atrophy is not the only response of motoneuron dendrites to axotomy, as thoracic motoneurons undergo a cyclic pattern of dendritic arbor degeneration and regeneration for at least 90 days following nerve crush [16]. Motoneurons innervating neck musculature in adult female cats show expanded dendritic arbors with growth cone-like structures appearing on some dendrites after axotomy [17]. The association of dendritic arbor size with muscle and functional synapse contact suggests that target muscles provide trophic support to motoneurons [18]. All these data suggest that PNIs that produce immediate denervation and (possible) reinnervation by slow axonal outgrowth also produce many changes in motoneuron morphology and CNS plasticities associated with loss and recovery of voluntary behaviors.

The current standard repair of single cut PNIs with a minimal gap between proximal and distal cut ends is to microsuture those ends (neurorrhaphy) with as little tension as possible. Larger gaps due to ablation PNIs are repaired by inserting acellular peripheral nerve conduits or cable autografts with viable Schwann and other non-neuronal cells to bridge the proximal and distal segments. Recovery from such ablation-type PNIs in more proximal limb segments, e.g., mid-thigh sciatic lesions, is typically poor to non-existent. Any behavioral recovery from single cut or ablation PNIs is often assumed to depend on successful regeneration of outgrowths from surviving proximal axonal ends to appropriately reinnervate denervated sensory or motor targets [19, 20]. In the absence of appropriate reinnervation, spinal connectivity can induce central plasticities to reorganize higher circuitry to compensate for inappropriate reinnervation [21]. For example, in nerve transfers, nearby healthy donor nerves are sutured to inappropriate muscles to restore innervation in the denervated muscle with no specificity of reinnervation. By decreasing reinnervation times, nerve transfers may increase CNS plasticities and relearning that gradually produce recovery of voluntary behaviors [22]. This capacity for relearning suggests that various CNS (or PNS) plasticities might alter spinal activation patterns after PNI to restore lost voluntary behaviors.

In contrast to PNI repair that depends on reinnervation by slow axonal outgrowth, we use a well-specified sequence of pharmaceutical solutions, one containing the plasmalemmal fusogen, polyethylene glycol (PEG) to non-specifically restore axolemmal and cytoplasmic continuity within seconds to minutes to many severed axons at each lesion site. This PEG-fusion repair at the cellular (axonal) level prevents the Wallerian degeneration of many severed distal axonal segments, maintains muscle innervation, and greatly improves behavioral recovery within weeks [23–27]. PEG-fusion has been used in primary nerve repair of single cut PNIs, as well as ablation-type PNIs repaired by PEG-fusion of viable donor peripheral nerve allografts from outbred Sprague-Dawley rats without tissue matching or immune suppression [23–27]. In both protocols, PEG does not specifically fuse the proximal/distal stumps of specific motor or sensory axons. That is, membranes in close approximation will PEG-fuse indiscriminately without regard for their original proximal-distal motor-sensory modality and/ or specificity. These inappropriate original connections must somehow be re-sorted by CNS/PNS plasticities in order to produce extensive voluntary behavioral recovery within 2–6 weeks post PEG-fusion repair.

In this study, we assess the ability of PEG-fusion to re-establish nerve continuity after rat sciatic nerve single cut or ablation PNIs and subsequent changes in motoneuron innervation pattern, soma size, dendritic arborization, and sensory primary projections that might have consequences for voluntary behavioral outcomes compared to standard microsuture repair (neurorrhaphy). We use cholera toxin B conjugated horseradish peroxidase to retrogradely label motoneurons projecting to the tibialis anterior (TA) muscle after sciatic PNIs to determine the extent to which PEG-fusion produces inaccurate, non-specific, reconnections of proximal-distal axons, as well the number and morphology of motoneurons correctly or incorrectly projecting to the TA. Our data show that nerve and muscle morphologies are similar after PEG-fusion repair of single cuts and allografts, and both exhibit greatly improved behaviors compared to negative controls that received all PEG-fusion solutions and protocols *except* PEG. Surprisingly, PEG-fusion of allografts produces significantly more cells labeled outside of the normal motoneuron pool, but does not diminish functional and behavioral recovery compared to single cuts with more accurate pairings with respect to unsevered control nerves. We propose that alterations in spinal connectivity and subsequent changes in motoneuron morphology are potential mechanisms for voluntary behavioral recovery after PEG-fusion, despite the lack of reinnervation specificity of axons when initially PEG-fused.

## Materials and methods

### Animals

**Fig 1:**
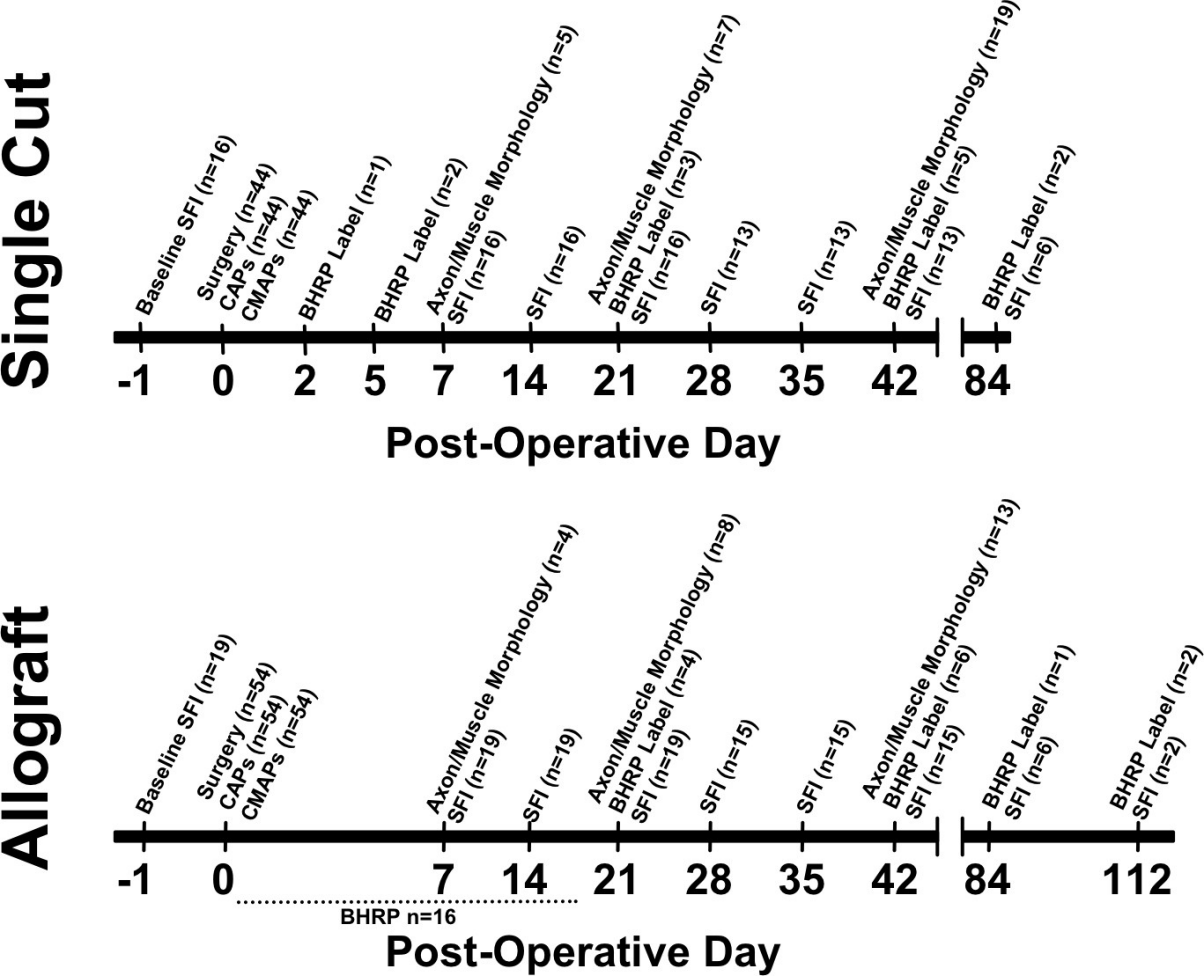
Experimental timeline. Timeline of experimental procedures for Single Cut (top) or Allograft (bottom) preparations. Surgery = Day of surgical operation. CAPs = Compound action potential recordings immediately post-operatively. CMAPs = Compound muscle action potential recordings immediately post-operatively. SFI = Sciatic Functional Index testing day. Axon/Muscle Morphology = Sciatic nerve and soleus muscle fibers examined for axon diameters, g ratio, soleus muscle fiber innervation and muscle fiber area. BHRP Label = Spinal cords examined for retrograde BHRP label in PEG-fused animals. Note that BHRP label was attempted, but not successful for PEG-Fused Allograft animals at 0-16d PO (n = 16).

All experimental procedures were approved by standards set forth by the Institutional Animal Care and Use Committee at the University of Texas at Austin. Rats of the same sex were housed 2-3/cage and maintained on a 12hr:12hr reverse light:dark cycle with food and water given ad libitum. Surgical and behavioral procedures were performed in the active cycle. For a detailed list of the timeline of various experimental procedures, see Fig 1.

### Surgical procedures

Young adult female Sprague–Dawley rats weighing 225 to 300 g were anesthetized with a small mammal anesthetic system for inhaled isoflurane (Handlebar Anesthesia, Austin, TX). After initial induction using a 4% isoflurane/oxygen mixture at a flow rate of 1.5 L/min, animals were maintained with a 1.5%–2% mixture at 1 L/min for the duration of the surgery. The lateral aspect of the left hindlimb was trimmed of fur and disinfected with 10% iodine/povidone. A 2 to 3 cm incision was made through the skin and the biceps femoris, parallel to its muscle fibers; muscle fibers were separated to expose the sciatic nerve. Animals were randomly assigned to PEG-fused or negative control groups after the neurorrhaphy to eliminate bias in surgical procedure. Negative control animals received neurorrhaphy repair and other solutions and procedures used for PEG-fusion repair, *except* the application of the PEG-solution [28]. Animals were administered carprofen (5.0mg/kg, s.c.) on the day of surgery, and every 24 hours for 72 hours thereafter. An additional group of age-matched, untreated animals served as unoperated controls.

### Single cut PEG repair

The left sciatic nerve was completely transected in the animal’s extracellular fluid with fine surgical scissors under sterile conditions. The nerve was then flushed with sterile Plasmalyte A (Baxter, Deerfield, IL), diluted in ddH_2_0 (250mOsm), a calcium-free hypotonic saline, to open cut ends and expel many intracellular vesicles that form to seal cut axonal ends. The severed and opened nerve ends were carefully trimmed and treated with an antioxidant (1% methylene blue; Acros Organics, Morris Plains, NJ) in sterile distilled water to keep axonal ends open and prevent formation of additional intracellular vesicles. The proximal and distal nerve segments were closely apposed with microsutures that provide mechanical strength at the lesion sites to prevent PEG-fused axons from pulling apart because axonal (or other cell-type) plasmalemmas have minimal tensile strength [28]. After this neurorrhaphy, a sterile hypotonic solution of 50% w/w PEG (Sigma Aldrich, St. Louis, MO; 3.35 kDa) in distilled water was applied to the repair site for 1–2 minutes to nonspecifically repair/join/fuse closely apposed open-cut axonal ends. The microsutured nerve ends were then irrigated several times with sterile isotonic Lactated Ringer’s containing calcium to repair axolemmal holes with calcium-induced vesicles. The incision wound was closed with sutures, and the animal was allowed to recover on heated pads. For details, see Ghergherehchi et al., 2019 [28].

### Double-transection ablation (Allograft) PEG repair

After exposure of the left sciatic nerve, 6-8mm segment was ablated mid-thigh with fine surgical scissors, leaving an 8-10mm gap between cut axonal ends in the proximal and distal stumps of the host nerve. Because intact nerves are under tension, an ablation produces a gap that is several millimeters longer than the removed segment. Donor Allografts that matched the diameter of the host nerve were obtained from the left or right sciatic nerve of another rat that was neither tissue matched nor immune-suppressed in either the donor or the host. Sciatic allografts were 1-3mm longer than the gap created by the ablated segment of the host nerve. Donor allografts were stored in calcium-free, hypotonic saline (Plasmalyte A) at 2°C for 30min–6h before use. Identical procedures for neurorrhaphy and PEG fusion described for Single cut repair were performed for the proximal and distal ends of the Allograft coaptation sites.

### Electrophysiological analyses

Stimulating and recording electrodes were placed approximately 1 cm apart across the site of intended single cut or ablation. Stimulation amplitudes were determined by identifying the stimulation voltage that generated maximum compound action potentials (CAPs) and compound muscle action potentials (CMAPs) response in intact nerves, and this voltage was maintained for stimulating CAPs and CMAPs after single cut or ablation and subsequent repair. A CAP was evoked by the rostral (stimulating) electrode and recorded by the distal electrode. The nerve was stimulated and CAPs recorded after severance and again after all lesion sites were microsutured and again after PEG-fusion for those animals receiving that treatment. CMAPs were evoked by stimulating the sciatic nerve in the upper thigh as described for CAPs and recording from the TA muscle. The stimulating electrode was placed proximal to any coaptation site, and a pair of monopolar needle electrodes were placed in the TA muscle. The nerve was stimulated and CMAPs recorded after severance and again after all lesion sites were microsutured and again after PEG-fusion for those animals receiving that treatment.

### Behavioral analyses

We used the Sciatic Functional Index (SFI) to assess hindlimb behaviors mediated by the sciatic nerve [29]. Animals were handled for acclimation and trained in the testing procedure for at least 1 week prior to surgery. Both hind paws were marked with red or blue ink, right side unoperated and left injured, respectively. The rat was then placed on an inclined 100-mm wide board, 5 feet in length, lined with paper strips. Runs without stops or hesitation producing three consecutive footprints of the left and right foot were scored and recorded, and two successful runs were collected for each rat per testing day [23, 24]. For both the normal and the experimental sides, assessors blinded to their treatment measured three variables for the normal and experimental footprints: footprint length, total toe spread, and intermediate toe spread as previously reported [30]. The mean of the six values for both the experimental and normal side from the two runs was recorded as the SFI score for the day. Animals were first tested 3 days after surgery, then weekly until the time of sacrifice (PEG-fused Single Cut, n = 10; PEG-fused Allograft, n = 13; Negative Control Single Cut, n = 6; Negative Control Allograft, n = 6).

### Axonal morphology

After a rat was deeply anesthetized with isoflurane and euthanized by cardiac KCl, sciatic nerves were removed from unoperated or experimental animals at 7, 21, or 42 days post-operatively (PO). The nerves were placed into 0.1 M sodium cacodylate buffer followed by 2% paraformaldehyde/3% glutaraldehyde fixative in buffer, or rats were perfused transcardially with buffer followed by fixative. Sciatic nerves and soleus muscles were pinned into Sylgard dishes with fixative overnight at room temperature. The next day, tissues were washed with buffer, trimmed and postfixed in 1% osmium tetroxide/1% potassium ferrocyanide in 0.1 M sodium cacodylate buffer for 3 to 5 hr, washed in water, stained in 1% aqueous uranyl acetate for 1 to 2 hr, washed and held in water [25, 26]. Segments of unoperated nerves used to compare with distal operated nerve segments were sampled in the distal third of the nerve, not including the peroneal–tibial bifurcation. Specifically, proximal and distal nerve segments were sampled 4 to 6 mm proximal and distal to the single cut, respectively. Much care was taken to ensure that sampling sites were equivalent in all nerves relative to suture placement.

Tissues were dehydrated though graded alcohols, exchanged to absolute acetone, placed in increasing concentrations of Hard Plus Resin 812 (Electron Microscopy Sciences, Hatfield, PA), and then embedded in fresh resin and polymerized at 60°C. Glass knife thick sections (0.5 μm) were stained in toluidine blue and examined for orientation and regions of interest with light microscopy prior to trimming for thin sectioning. Thin sections (silver‐gold, 65 nm) were cut on a diamond knife (DDK, Wilmington, DE) and mounted on formvar‐coated Synaptek grids (Electron Microscopy Sciences, Hatfield, PA) prior to viewing in a Technai Spirit electron microscope (Hillsboro, OR) fitted with an AMT Advantage HR 1kX1k digital camera. Neuromuscular junctions (NMJs) and multiple regions within and around sciatic nerves were imaged for further analyses.

For axon morphology, 10 to 20 electron micrographs per nerve were taken at 1700×. To avoid bias, all intact *en face* axons in an image were measured until at least 100 to 150 were recorded, except for some negative control animals for which < 100 axons could be counted in many images. Individual axons were excluded if they exhibited redundant or separated myelin layers, myelin infolds that crossed the axon midline, or electron‐dense axoplasm. Axon diameter was calculated as (√axon area/π)*2, fiber diameter (diameter of axon+myelin sheath) was calculated as (√fiber area/π)*2, and g ratio was calculated as axon diameter/diameter of axon+myelin sheath.

### Muscle fiber innervation and area

After rats were deeply anesthetized with isoflurane and euthanized by cardiac KCl, soleus muscles were taken from unoperated rats and the operated sides of PEG‐fused or negative control rats at 7, 21 and 42d PO. Muscles were dissected into phosphate‐buffered saline (PBS) and pinned into Sylgard‐coated dishes prior to fixation and permeabilization in −20°C methanol, then rehydrated and washed in PBS [31]. Muscles were placed in microcentrifuge tubes and blocked with 0.2% BSA, 0.3% TritonX‐100 in PBS with 0.1% Azide for 30 min. SV2 (anti‐synaptic vesicle protein 2, Developmental Studies Hybridoma Bank) and 2H3 (anti‐neurofilament, DSHB) were added (1:400 each in blocking solution), and the muscles were maintained overnight at 4°C. Muscles were then washed in blocking solution and incubated in anti‐mouse Alexa 488 (1:600, Molecular Probes, Invitrogen) for 1 hr at room temperature. Bungarotoxin conjugated to Alexa 647 (1:800, Molecular Probes, Invitrogen) was used to label the acetylcholine receptors (AChRs) prior to washes in PBS. Thin sheets of muscle fibers were dissected from the muscle surface and mounted on glass slides with Permafluor (Thermo Scientific). NMJs were observed with a Zeiss Axiovert 200M (Pleasanton, CA). Labeled axons parfocal and in contact with a receptor were considered to innervate the receptor. Confocal images were captured on a Zeiss LSM 710 with a 40 × oil immersion lens and compressed *z*‐stacks of images prepared in ImageJ.

To measure muscle fiber area, animals were perfused with 4% paraformaldehyde and kept in the same fixative overnight. After embedding, thick plastic sections (0.5 μm) from NMJ regions where the nerve inserts at the belly of soleus muscles were imaged. At least 100 muscle fibers for each muscle were traced in ImageJ, and the measurements were recorded.

### Spinal cord histochemical and histological processing

To determine the timing of the re-establishment of retrograde transport and the specificity of connections made by PEG-fused maintained axons, we used cholera toxin B conjugated horseradish peroxidase (BHRP; Invitrogen, Carlsbad, CA). BHRP labeling permits population-level quantitative analysis of motoneuron soma and dendritic morphologies [32]. Animals that received axotomy with PEG-fusion repair (0d-112d PO) or negative control (2d-42d PO) were anesthetized with isoflurane; BHRP (0.5μl, 0.2% in distilled water) was injected bilaterally into the TA to label motoneurons by fast retrograde transport. Unoperated control animals received similar injections into either the TA or the FDB muscles. At this volume and concentration, BHRP is specifically taken up by distal motor axons and does not label dorsal root ganglion cells that lack GM1 ganglioside required to actively transmembrane transport BHRP [33, 34].

Forty-eight hours after BHRP injection, a period that ensures optimal labeling of motoneurons [32], animals were anesthetized with isoflurane, euthanized by intraperitoneal injection of Euthasol, and perfused intracardially with saline followed by cold fixative (1% paraformaldehyde/1.25% glutaraldehyde). The lumbar portion of the spinal cord of each animal was removed, postfixed in the same fixative for 5 hours, and then transferred to sucrose phosphate buffer (10% w/v, pH 7.4) overnight for cryoprotection. Spinal cords were embedded in gelatin, frozen, and sectioned transversely at 40 μm; all sections were collected into four alternate series. One series was stained with thionin for use in motoneuron counts and measurement of soma size. For visualization of BHRP, the three remaining series were immediately reacted using a modified tetramethyl benzidine protocol [32], mounted on gelatin-coated slides, and counterstained with thionin.

### Spinal cord labeling

For each animal, the entire rostrocaudal range of spinal segments contributing to the sciatic nerve was examined under darkfield illumination. We noted the spinal segment, location in the gray matter, and nature of BHRP labeling, including sensory afferent labeling in the dorsal horn, consisting of (1) labeled fibers, (2) large diffusely distributed granules of reaction product characteristic of terminal labeling, or (3) motoneuron labeling, consisting of labeled somata and associated dendritic arbors. Spinal segment boundaries were determined using the midpoints between dorsal root entrances [35].

### Motoneuron number and morphology

We assessed two different motor populations that contribute to the sciatic nerve, motoneurons innervating the TA of the lower leg and those innervating the FDB, an intrinsic foot muscle. These motor populations were selected because they represent the extremes of the proximal-distal innervation of the sciatic nerve, and consequently, the extremes of the rostrocaudal spinal segment locations of the contributing motoneuron populations.

### Motoneuron counts

The TA muscle is innervated by motoneurons located in column 4 of the lateral motor column in the L3 spinal segment [36]. Motoneurons innervating the TA muscle do not form a discrete nucleus, but instead are contained within the large continuous populations of motoneurons located within the lateral motor column. To identify the appropriate area within the lateral motor column for motoneuron counts in the unreacted series, we used a method similar to that of Little et al. [37]. Briefly, for each animal, the range of sections in which motoneurons labeled with BHRP after injection into the right (control side) TA muscle were present in the reacted series was identified. Motoneurons were counted in the appropriate matching sections in the unreacted series. For each animal, estimates of the total number of motoneurons in the left and right lateral motor columns were obtained using the optical disector method using Stereo Investigator (MBF Bioscience, Williston, VT) as previously described [37]. Counts were made at x937.5 under brightfield illumination, where motoneurons are easily recognizable as large, darkly staining, multipolar cells.

A counting frame (110 μm x 80 μm) was moved systematically throughout an area of each ventral horn (~500 μm x 500 μm, defined by the actual distribution of BHRP-labeled somata in unoperated animals) in each section within the identified range. Only motoneurons in which there was a clear nucleus and nucleolus were counted, provided they did not contact the forbidden lines of the counting frame; motoneuron nucleoli were counted as they appeared while focusing through the z-axis, and nucleoli in the first focal plane (i.e., “tops”) were excluded to avoid double counting. The length of the disector was approximately 16 μm, which was adequate for visualizing nucleoli in multiple focal planes. Motoneuron counts were derived from a mean of 5.3 sections spaced 480 μm apart and distributed uniformly through the entire rostrocaudal extent of the TA motoneuron pool range. This sampling scheme produced a mean estimated coefficient of error (CE) of 0.05. Cell counts for each animal were corrected for the proportion of sections sampled, and then expressed as a ratio (motoneuron number on the experimental side relative to that on the intact side) to quantify the magnitude of potential motoneuron loss (Unoperated, n = 7; PEG-fused Single Cut, n = 8; PEG-fused Allograft, n = 24; Negative Control Single Cut, n = 5; Negative Control Allograft, n = 3).

The FDB muscle is innervated by motoneurons located in the L5/L6 spinal segments [36, 38]. Unlike TA motoneurons, FDB motoneurons are located in a discrete nucleus, the retrodorsal lateral nucleus (RDLN) [39]. Motoneurons in the RDLN were counted in the unreacted series using methods similar to those described above. Motoneuron counts were derived from a mean of 4.2 sections spaced 480 μm apart; this sampling scheme produced a mean estimated coefficient of error (CE) of 0.04 (unoperated, n = 11; PEG-fused Single Cut, n = 17; PEG-fused Allograft, n = 25; Negative Control Single Cut, n = 4; Negative Control Allograft, n = 3).

### Soma size

Sections from the unreacted series were used for measuring motoneuron soma size using the Nucleator method [40]. A set of 4 rays emanating from a point randomly chosen within each Nissl-stained motoneuron soma was drawn and oriented randomly. Soma areas from a mean of 38.6 ± 2.7 TA and 27.7 ± 1.2 RDLN motoneurons were measured for each animal using a video-based morphometry system (Stereo Investigator; MBF Bioscience, Williston, VT) at a final magnification of X780. The overall mean estimated coefficient of error (CE) for TA motoneurons was 0.06, and for RDLN motoneurons was 0.01. Soma areas for each motor pool within each animal were then averaged for statistical analysis (TA: Unoperated, n = 12; PEG-fused Single Cut, n = 12; PEG-fused Allograft, n = 21; Negative Control Single Cut, n = 5; Negative Control Allograft, n = 3; RDLN: Unoperated, n = 11; PEG-fused Single Cut, n = 17; PEG-fused Allograft, n = 25; Negative Control Single Cut, n = 2; Negative Control Allograft, n = 2).

Soma sizes of BHRP-labeled motoneurons were also measured using similar methods. Soma areas from a mean of 22.0 ± 2.3 BHRP-labeled motoneurons were measured for each animal (mean CE = 0.025; Unoperated TA, n = 9; Unoperated FDB, n = 5; PEG-fused Single cut, n = 13; PEG-fused Allograft, n = 9; Negative Control Single Cut, n = 1; Negative Control Allograft, n = 1).

### Dendritic length

For each animal examined, dendritic lengths in a single representative set of alternate series were measured under darkfield illumination (Unoperated TA, n = 12; Unoperated FDB, n = 7; PEG-fused Single Cut d, n = 13; PEG-fused Allograft, n = 10). Beginning with the first section in which BHRP-labeled fibers were present, labeling through the entire rostrocaudal extent of the L3/L4 and L5/L6 spinal segments was assessed. BHRP-labeled fibers were traced in every third section (480 μm apart) in three dimensions using a computer-based morphometry system (Neurolucida, MBF Bioscience, Williston, VT) at a final magnification of x250. For each animal, mean dendritic length per labeled motoneuron within each of the L3/L4 and L5/L6 spinal segments was estimated by creating a composite of all sections within the respective segments, summing the measured dendritic lengths within those segments, multiplying by three to correct for sampling, then dividing by the total number of labeled motoneurons present. Counts of labeled motoneurons were made under brightfield illumination, where somata could be visualized and cytoplasmic inclusion of BHRP reaction product confirmed. This method does not attempt to assess the actual total dendritic length of labeled motoneurons [41], but it has been shown to be a sensitive and reliable indicator of changes in dendritic morphology after a variety of experimental manipulations (e.g., [37, 32, 42, 43]).

### Dendritic distribution

Motoneuron dendritic distributions normally differ across motor populations that innervate different target musculature. To assess potential redistributions of motoneuron dendrites within the L3/L4 and L5/L6 spinal segments across treatment groups, for each animal the composite dendritic arbor created in the length analyses was divided using a set of axes oriented radially around the center of the collective labeled somata. These axes divided the spinal segments into twelve bins of 30° each. The portion of each animal’s dendritic arbor per labeled motoneuron contained within each location was then determined. This method provides a sensitive measure of dendritic redistribution in response to changes in afferent input (e.g., [43, 44]).

### Statistical analysis

All data were analyzed by t-test, analyses of variance (one way or repeated measures as appropriate), followed by *post hoc* analyses using Fisher’s least significant difference (LSD), or simple regression. Because some of the animals in the study could not be included in all analyses, group sizes for each analysis are reported individually below (in every case the reported n represents the number of animals included in that analysis). For all data, values are reported as Mean ± SEM. Digital light micrographs were obtained with an MDS 290 digital camera system (Eastman Kodak Company, Rochester, NY). Brightness and contrast of these images were adjusted in Adobe Photoshop (Adobe Systems, San Jose, CA).

## Results

### Electrophysiological function

Electrical stimulation of intact sciatic nerves produces action potential propogation to the spinal cord and to distal targets, resulting in sensory perceptions and contraction of hindlimb muscles [1]. CAPs are evoked action potentials extracellularly recorded from nerve axons and are used to assess electrophysiological and axonal continuity [27, 45]. CMAPs are evoked action potentials extracellularly recorded from a set of muscle fibers and are used to test muscle activation in response to nerve stimulation. We regard CAPs and CMAPs as a “yes/no” (binary) measure because their amplitude and time course depends on many variables, including electrode shape, construction and placement [24].

**Fig 2:**
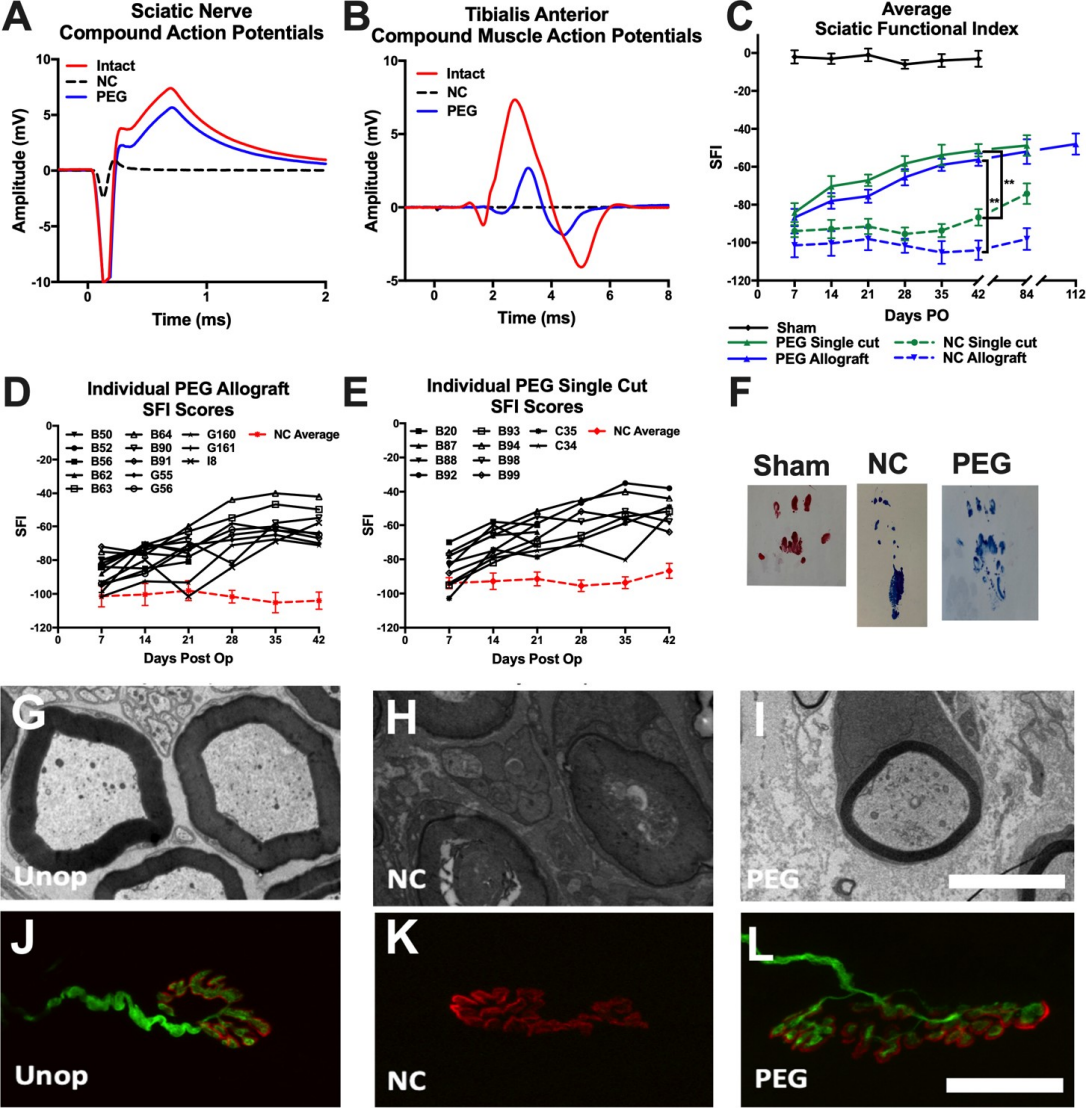
Electrophysiological, functional, and morphological results of PEG-fusion repair. Representative electrophysiological recordings of **(A)** sciatic nerve compound action potentials (CAPs) or **(B)** compound muscle action potentials (CMAPs) in intact nerves (red line), after microsuture (neurorrhaphy) repair without PEG application (Negative Control; black line) or with PEG-fusion repair (PEG; blue line). **(C)** Sciatic Functional Index (SFI) scores for Sham Operated animals (black line, n = 4), and Single Cut (green lines, n = 16), and Allograft (blue lines, n = 19) neurorrhaphy repair animals with or without PEG-fusion. **p < 0.*01*. **(D)** Individual SFI scores for PEG-fused Allograft animals (n = 13) or mean SFI score for Negative Control Allograft animals (n = 6, red line). **(E)** Individual SFI scores for Single Cut PEG-fusion animals (n = 10) or mean SFI score for animals Negative Control Single Cut animals (n = 6, red line). **(F)** Representative footprints for Sham Control (left), Negative Control Single Cut (middle) and PEG-fused Single Cut animals (right). **(G-I)** TEM cross sections of **(G)** Unoperated Control sciatic axons, **(H)** Negative Control Single Cut (NC) axons and **(I)** PEG-fused Single Cut axons and in the distal nerve segment. Scale bar = 5 μm. **(J-L)** Soleus muscle fiber innervation of **(J)** Unoperated Control (unop), **(K)** Negative Control Single Cut, and **(L)** PEG-fused Single Cut neuromuscular junctions. Scale bar = 30 μm.

Our stimulation of intact sciatic nerves (n = 98) always produced CAPs recorded from the nerve or CMAPs recorded from the TA muscle (Fig 2A and 2B) or other sciatic-innervated muscles. After a single cut of the sciatic nerve, stimulation of the proximal nerve segment failed to elicit distal nerve CAPs or CMAPs from the TA. (Stimulation of a singly cut sciatic nerve sometimes evokes CMAPs or twitches in distal muscle masses by collateral branches that occasionally arise proximal to the proximal stimulating electrodes [46]. These CMAPs are no longer observed if the collateral branch is subsequently also transected.) CAPS and CMAPs were not restored after microsuture repair in Negative Control Single Cut nerves (n = 13), i.e., if the PEG-solution was not applied, axonal continuity was not restored by neurorrhaphy alone (Fig 2A and 2B). If PEG was applied after neurorrhaphy in PEG-fused Single Cut nerves (n = 31), CAP propagation was immediately re-established from the proximal segment, across the lesion site into the distal nerve segment to activate NMJs. Transmitter release at NMJs produced CMAPS, such as those recorded from the TA muscle that produced TA muscle contraction, i.e., complete functional reinnervation without fully restoring TA coordinated use in voluntary behaviors as described in Fig 2C and subsequent section on locomotor behaviors. That is, PEG-fused axons were anatomically continuous with nerve-muscle functions restored from proximal to any lesion site, across the PEG-fused single cut site, to distal axonal segments that innervate and activate distal targets.

Sciatic nerve ablations repaired by donor allografts that were microsutured to the host proximal and distal axonal segments (Negative Control Allografts) also exhibited disrupted axonal continuity (n = 11). Stimulation of the proximal segment failed to elicit CAPs recorded from the donor nerve segment or the distal host nerve (Fig 2A), or CMAPs in the TA muscle (Fig 2B), i.e., axons were not continuous across the proximal and/or distal lesion sites. PEG-fusion of donor allografts (PEG-fused Allografts) restored CAP conduction from the proximal segment across the donor graft and throughout the distal host segment (Fig 2A) to produced CMAPs in the TA muscle (Fig 2B) and twitches of the TA and other hindlimb muscles (n = 43). That is, PEG-fused axons in donor allograft repairs were anatomically continuous with functions restored from proximal across the donor allografts to sites to distal host axonal segments that innervate and activate distal targets.

### Locomotor behaviors

The SFI test relies on the difference in various measures of gait between the operated and unoperated hindlimb [30]. Recovery, if any, after standard neurorrhaphy of single cuts or ablations is due to axons regenerating by outgrowth from severed surviving proximal axonal segments that may increasingly reinnervate denervated muscles in a more-proximal to more-distal sequence.

In our Sham Operated animals (n = 4), footprints for the right and left hindlimb typically had similar values for footprint length, total toe spread, and intermediate toe spread, resulting in SFI scores of -4.0 ± 3.3, with no significant change over time (Fig 2C and 2F). Microsuture repair of Negative Control Single Cut nerves (n = 6) produced asymmetrical hindlimb locomotive patterns, such that the operated hindlimb exhibited an increase in footprint length and a decrease in toe spread (Fig 2F), with SFI scores of -93.9 ± 3.2 at 7d PO associated with complete loss of sciatic-mediated voluntary behaviors. SFI scores gradually increased to -86.6 ± 4.3 at 42d PO, and significantly increased to -74.2 ± 5.4 by 84d PO [t(8) = 3.3, *p* < 0.01]. In contrast, rats with PEG-fused Single Cut nerves (n = 10) had some sciatic-mediated behaviors restored at 7d PO (SFI = -84.1 ± 2.9) followed by significant increases in SFI scores that plateaued at -51.2 ± 3.2 at 42d PO. These animals gradually recovered the ability to lift their foot and spread toes during locomotion (Fig 2F). PEG-fused Single Cut animals had higher SFI scores compared to Negative Control Single Cut preparations starting at 14d PO [t(14) = 2.8, *p* < 0.05], and continued to increase at all times sampled up to 42d PO [t(11) = 6.6, *p* < 0.001]. Overall, PEG-fusion repair of single cuts produced more complete recovery compared to microsuture repair of Negative Control Single Cut nerves [F(1,5) = 6.0, *p* < 0.01]. Due to the small sample size of PEG-fused Single Cut animals at 84d PO (n = 2), statistical comparisons were not made for this time point.

Animals with ablation injuries 8-10mm in length repaired with 10 -12mm long donor sciatic allografts (n = 6) exhibited immediate loss of SFI functions (mean = -101.4 ± 6.3), with no significant recovery in voluntary SFI behaviors at 42d PO (-104.2 ± 5.1; *p* > 0.05) or up to 84d PO (-98.1 ± 5.7), i.e., complete loss of sciatic-mediated behaviors at all tested PO times. PEG-fused Allografts (n = 13) showed some return of sciatic-mediated behaviors at 7d PO (SFI = -86.7 ± 4.5) that increased to plateau at 42d PO (-56.2 ± 3.2). SFI scores of PEG-fused Allograft animals were significantly higher than Negative Control Allografts starting at 14d PO [t(17) = 2.9, *p* < 0.01], and continued to produce more complete behavioral functions at all times tested up to 42d PO [t(13) = 8.3, *p* < 0.001]. PEG-fused Allografts produced more complete recovery compared to Negative Control Allografts [F(1,5) = 5.6, *p* < 0.01], and was similar to the behavioral recovery seen for PEG-fused Single Cut animals [F(1,5) = 0.71, *ns*]. SFI scores of Negative Control Single Cuts were significantly higher than those of Negative Control Allografts at 84d PO [t(5) = 2.9, *p* < 0.05], indicating that single cut injuries could recover some sciatic voluntary behaviors after neurorrhaphy by 84d PO, but Negative Control Allografts receiving only neurorrhaphy did not exhibit any recovery of voluntary sciatic behaviors from 0-84d PO. Due to the small sample size of PEG-fused Allograft animals at 84-112d PO (n = 3), statistical comparisons were not made for these time points.

### Nerve and muscle morphology

Axons of the sciatic nerve were assessed by TEM cross sections distal to the lesion site. Sciatic nerves in Unoperated Control Animals showed normal axon morphology [25, 26], with axonal diameters of 3.82 ± 1.64 μm and g ratios of myelin sheaths of 0.61 ± 0.06 (Fig 2G, Table 1). Negative Control Single Cut nerves receiving only microsuture repair showed Wallerian degeneration of all axons distal to the lesion site by 7d PO (Fig 2H). PEG-fusion repair of Single Cut nerves prevented Wallerian degeneration of many, but not all, axons in distal axonal segments (Fig 2I), with mean axon diameters of 1.85 ± 0.78 μm at 7d PO that increased to 2.69 ± 1.19 μm at 42d PO (Table 1). Small diameter axons (1.72 ± 0.44 μm) regenerating by outgrowths from severed proximal segments in Single Cut Negative Control nerves were observed in the distal segment at 21d PO, and increased in diameter (1.90 ± 0.83 μm) by 42d PO (Table 1). Axon diameters were larger in Single Cut animals that received PEG-fusion compared to Negative Controls at all PO times (Table 1).

**Table 1:** Behavioral and peripheral morphological measures.

Group	SFI	Distal axon diameter (μm)	g ratio, distal	MFI (%)	MFA (μm2)
**Unoperated**					
UNOP	-4 ± 3.3	3.82 ± 1.64	0.61 ± 0.06	>99 ± 1%	2710 ± 710
**7d PO**					
PEG SC	-72 ± 6.2	1.85 ± 0.78	0.68 ± 0.07	80 ± 9%	
PEG ALLO	-78.2 ± 6.7	4.22 ± 1.82	0.63 ± 0.04	71 ±15%	
NC SC	-95.5 ± 2.6	0 ± 0	0 ± 0	0 ± 0%	1370 ± 470
NC ALLO	-99. 3 ± 6.9	0 ± 0	0 ± 0	0 ± 0%	
**21d PO**					
PEG SC	-55.4 ± 5.5	4.47 ± 2.66	0.71 ± 0.08	78 ± 38%	
PEG ALLO	-46.9 ± 12.9	2.01 ± 0.99	0.62 ± 0.08	86 ± 7%	1859 ± 1070
NC SC	-91.4 ± 4.0	1.72 ± 0.44	0.76 ± 0.05	0 ± 0%	
NC ALLO	-98.0 ± 5.8	1.35 ± 0.22	0.78 ± 0.07	0 ± 0%	620 ± 210
**42d PO**					
PEG SC	-41.3 ± 5.5	2.69 ± 1.19	0.64 ± 0.08	97 ± 3%	1780 ± 710
PEG ALLO	-53.1 ± 6.1	2.85 ± 1.71	0.67 ± 0.08	99 ± 1%	2360 ± 1100
NC SC	-87.3 ± 5.5	1.90 ± 0.83	0.63 ± 0.08	46 ± 21%	1440 ± 500
NC ALLO	-106.0 ± 4.7	1.59 ± 0.62	0.63 ± 0.10	8 ± 2%	790 ± 260

Rows give means ± SD of behavioral and morphological measures at 7, 21, or 42d PO for the following groups: Unoperated Controls (UNOP), PEG-fused Single Cut (PEG SC), PEG-fused Allograft (PEG ALLO), Negative Control Single Cut (NC SC) and Negative Control Allograft (NC ALLO). Columns show mean values for SFI scores, distal axon diameter (μm), g ratio (axon diameter/diameter of axon+myelin sheath), % of muscle fibers having innervated soleus NMJs (MFI), and soleus muscle fiber area (MFA).

As reported for Single Cut PEG-fused sciatic nerves, axonal diameters in PEG-fused Allografts were maintained at all PO times (Table 1), with axon diameters (4.22 ± 1.82 μm) at 7d PO, and (2.85 ± 1.71 μm) at 42d PO (reduced mean diameter was due in part to regenerating axons). Negative Control Allografts that lacked PEG-fused axons exhibited complete degeneration of all axons in the distal segment by 7d PO (Table 1), with small regenerating fibers (1.59 ± 0.62 μm) observed in the distal segment by 42d PO (Table 1).

Muscle fiber innervation was assessed from soleus and TA muscles using fluorescence and confocal immunohistochemistry. Unoperated Control animals showed normal neuromuscular innervation (>99 ± <1%) of both soleus and TA muscles (Fig 2J, Table 1). Standard microsuture repair of Negative Control Single Cut nerves produced complete denervation at 7d PO (Fig 2K, Table 1). Animals with PEG-fused Single Cut repair had 80 ± 9% innervation at 7d PO of soleus muscle fibers (Fig 2L) whose innervation increased to 97 ± 3% at 42d PO (Table 1). At 42d PO in Negative Control Single Cuts, some axons regenerating by outgrowth innervated NMJs (46 ± 21%). PEG-fused Allografts similarly preserved NMJ innervation (Table 1) at all PO times, with 71 ± 15% innervation at 7d PO that increased to 99 ± 1% at 42d PO. Negative Control Allografts that were not PEG-fused exhibited complete denervation of NMJs by 7d PO, with few NMJs (8 ± 2%) innervated by 42d PO (Table 1).

In Unoperated Control animals, soleus muscle fibers showed normal area [25, 26] of 2710 ± 710 μm^2^ (Table 1). Negative Control Single Cut animals had reduced muscle fiber area (1440 ± 500 μm^2^) at 42d PO, while PEG-fused Single Cut animals showed reduced muscular atrophy (1780 ± 710 μm^2^) at the same sampling time. Negative Control Allograft animals had muscle fiber areas of 790 ± 260 μm^2^ at 42d PO, while PEG-fused Allograft animals had muscle fiber areas of 2360 ± 1100 μm^2^. Negative Control Allografts had smaller muscle fiber area compared to Negative Control Single Cuts, consistent with our data showing fewer axons innervating these muscle fibers.

These results showed that single cut and ablation repairs using neurorrhaphy without PEG-fusion produced complete degeneration of distal axons, atrophy of muscle fibers, and denervation of NMJs by 7d PO. Negative Control Single Cut nerves have some axons that regenerate by outgrowth to eventually innervate NMJs. Negative Control Allografts have fewer, smaller diameter, axons regenerating by outgrowth that innervate fewer NMJs by 42d PO (Table 1). In contrast, PEG-fusion repair of single cuts and allografts maintained axonal diameters, reduced muscular atrophy, and preserved NMJ innervation at 42d PO—and at all other PO times examined in this study.

### Motoneuron numbers

**Fig 3:**
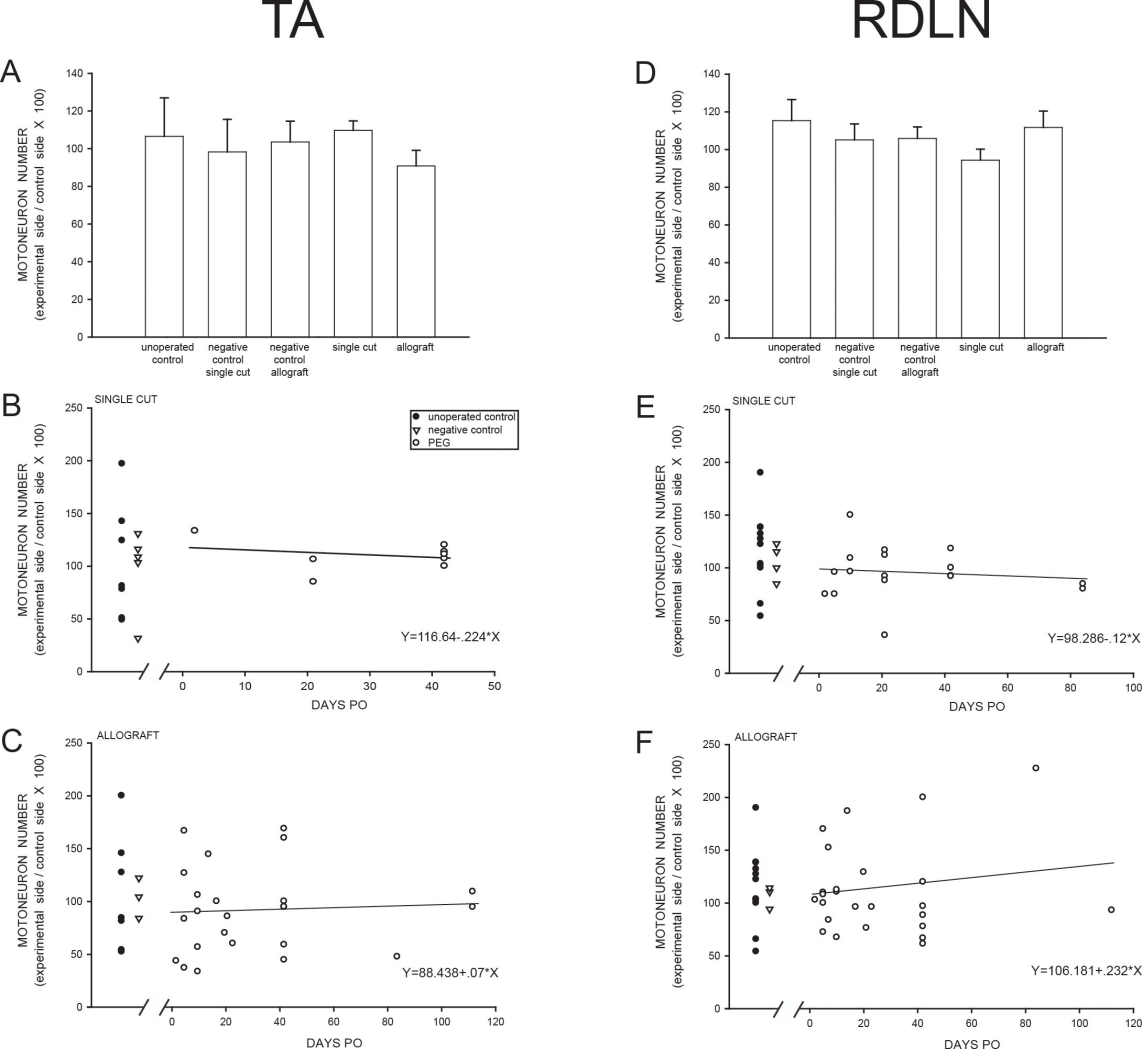
Motoneuron number. Mean numbers (pooled over all post-operative times) of TA **(A)** and RDLN **(D)** motoneurons in Unoperated Control, Negative Control, Single cut PEG-repair, and Allograft PEG-repair animals expressed as a ratio of motoneuron number on the experimental (left) side relative to that on the unoperated (right) side. Bar heights represent means ± SEM. Numbers of TA or RDLN motoneurons in PEG-fused animals do not significantly vary by PO survival times for Single cut **(B, E)** or Allograft animals **(C, F)**. Individual animals indicated by filled circles, Unoperated Controls; open triangles, Negative Controls; open circles, PEG-fused animals.

In Unoperated animals, the number of Nissl-stained motoneurons in the TA motor pool did not differ between the left (503.3 ± 52.0) and right motor column (539.8 ± 70.6) [paired t-test, t(6) = -0.37, *ns*; left/right ratio = 106.3 ± 20.4] (Table 2). Axotomy with or without subsequent PEG-fusion had no effect on the mean ratio [F(4,42) = 0.49, *ns*] of TA motoneurons on the experimental side (left) relative to the intact side (right) (PEG-fused Single Cut left/right ratio = 109.5 ± 5.0; PEG-fused Allograft left/right ratio = 90.6 ± 8.3; Negative Control Single Cut left/right ratio = 98.1 ± 17.3; Negative Control Allograft left/right ratio = 103.3 ± 11.0;Table 2, Fig 3A]. The number of TA motoneurons in PEG-fused Single Cut or Allograft animals also did not vary by PO survival times, with no significant correlation between left/right ratios and days PO after either Single cuts [F1,7) = 0.37, *ns*] or Allografts [F(1,23) = 0.07, *ns*] (Fig 3B and 3C).

**Table 2:** Motoneuron number and morphological characteristics.

	Motoneuron Number		Soma Size (μm^2^)		Soma Size (μm^2^)		Dendritic Length (μm)	
Group	[experimental side (left) / control side (right)] x 100		(Nissl)		(BHRP)			
	TA	RDLN	TA	RDLN	TA	RDLN	TA	RDLN
UNOP	106.3 ± 20.4	115.9 ± 11.2	925.9 ± 15.3	731.2 ± 40.4	975.4 ± 42.9	744.3 ± 39.6	4023.7 ± 604.7	2265.4 ± 336.6
PEG SC	109.5 ± 5.0	94.9 ± 5.8	923.6 ± 40.4	746.5 ± 38.3	849.2 ± 36.5	779.3 ± 80.1	5071.3 ± 1037.5	5136.4 ± 1642.8
PEG ALLO	90.6 ± 8.3	112.3 ± 8.7	905.5 ± 44.0	725.1 ± 27.4	834.3 ± 69.0	787.8 ± 48.5	2887.3 ± 734.8	2615.2 ± 1175.9
NC SC	98.1 ± 17.3	105.7 ± 8.4	992.0 ± 50.7	639.4 ± 32.6	870.0 ± 106.4	630.1 ± 28.1	*	*
NC ALLO	103.3 ± 11.0	106.4 ± 6.1	897.7 ± 93.0	658.7 12.7	799.3 ± 71.2	908.4 ± 71.2	*	*

Rows give means ± SEM of motoneuron number and morphological measures for motoneurons located in column 4 of the lateral motor column in the L3 spinal segment that innervate the tibialis anterior (TA) or located in the retrodorsal lateral nucleus (RDLN) that innervate the flexor digitorum brevis from the following groups: Unoperated Controls (UNOP), PEG-fused Single Cut (PEG SC), PEG-fused Allograft (PEG ALLO), Negative Control Single Cut (NC SC) and Negative Control Allograft (NC ALLO). Columns show mean values for motoneuron number expressed as a ratio (motoneuron number on the experimental side relative to that on the intact side) to quantify the magnitude of potential motoneuron loss, soma size from both Nissl-stained as well as BHRP-labeled material, and dendritic length per labeled motoneuron.

* Labeling of motoneuron somata and proximal dendrites was rarely present in Negative Control animals, and was too faint to permit dendritic reconstruction.

In Unoperated Control animals, the number of Nissl-stained motoneurons in the RDLN did not differ between the left (393.8 ± 25.2) and right motor column (357.8 ± 22.9) [paired t-test, t(10) = 0.98, *ns*; left/right ratio = 115.9 ± 11.2] (Table 2). Axotomy with or without subsequent PEG-fusion had no effect on the mean ratio [F(4,55) = 0.80, *ns*] of RDLN motoneuron number on the left side of Single Cut or Allograft animals relative to the intact side (right) (PEG-fused Single Cut left/right ratio = 94.9 ± 5.8; PEG-fused Allograft left/right ratio = 112.3 ± 8.7; Negative Control Single Cut left/right ratio = 105.7 ± 8.4; Negative Control Allograft = 106.4 ± 6.1; Table 2, Fig 3D]. The number of RDLN motoneurons in PEG-fused Single Cut or Allograft animals also did not vary with PO survival times, i.e., no significant correlation between left/right ratio and days PO in either PEG-fused Single Cut [F(1,15) = 0.24, *ns*] or PEG-fused Allograft sciatic nerve [F(1,23) = 0.48, *ns*] animals (Fig 3E and 3F).

### Soma size

#### Nissl-stained somata

**Fig 4:**
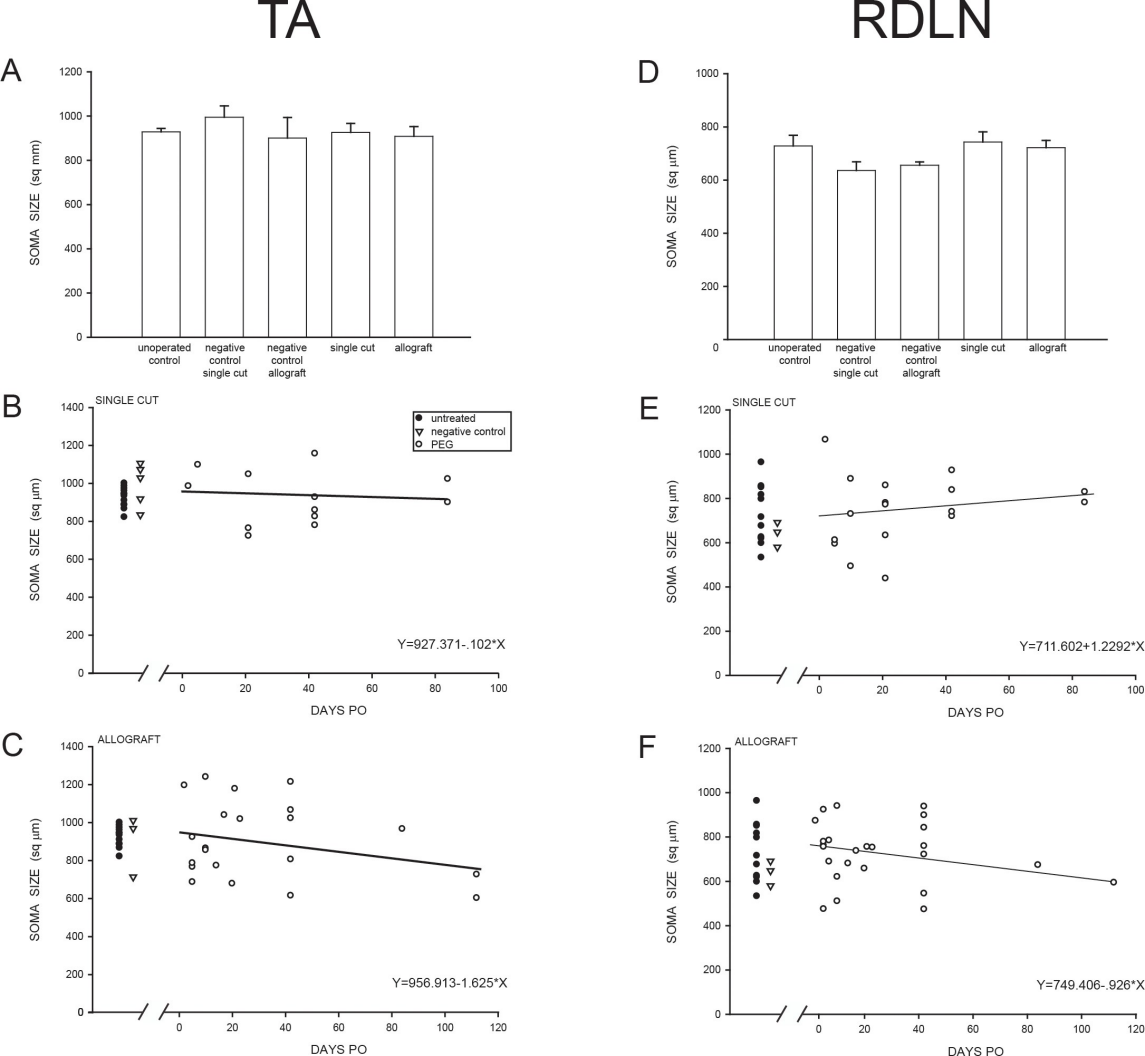
Soma size. Mean soma sizes (pooled over all post-operative times) of Nissl-stained TA **(A)** and RDLN **(D)** motoneurons in Unoperated Control, Negative Control, PEG-fused Single Cut and PEG-fused Allograft animals. Bar heights represent means ± SEM. Soma size of TA or RDLN motoneurons does not significantly vary by PO survival times in PEG-fused Single Cut **(B, E)** or Allograft animals **(C, F)**. Individual animals indicated by filled circles, Unoperated Controls; open triangles, Negative Controls; open circles, PEG-fused animals.

In Unoperated Control animals, the mean cross sectional area of TA motoneuron somata was 925.9 ± 15.3 μm^2^ (Table 2), and did not differ from the mean soma size on the PNI lesion side of Single Cut or Allograft animals with or without subsequent PEG-fusion [F(4,48) = 0.33, *ns*; PEG-fused Single Cut, 923.6 ± 40.4 μm^2^; PEG-fused Allograft, 905.5 ± 44.0 μm^2^; Negative Control Single Cut, 992.0 ± 50.7 μm^2^; Negative Control Allograft, 897.7 ± 93.0 μm^2^; Table 2, Fig 4A]. Furthermore, soma size of TA motoneurons in PEG-fused Single Cut or Allograft animals did not vary with PO survival time, i.e., no significant correlation between soma size and days PO in either Single Cut [F(1,11) = 0.004, *ns*] or Allograft [F(1,20) = 1.47, *ns*] animals (Fig 4B and 4C).

In Unoperated Control animals, the mean cross sectional area of RDLN motoneuron somata was 731.2 ± 40.4 μm^2^ (Table 2), and did not differ from the mean soma size on the PNI side of Single Cut or Allograft animals with or without subsequent PEG-fusion [F(4,54) = 0.57, *ns*; PEG-fused Single Cut, 746.5 ± 38.3 μm^2^; PEG-fused Allograft, 725.1 ± 27.4 μm^2^; Negative Control Single Cut, 639.4 ± 32.6 μm^2^; Negative Control Allograft, 658.7 ± 12.7 μm^2^; Table 2, Fig 4D]. Soma size in Negative Control Single Cut or Allograft animals was non-significantly (13% and 10%, respectively) smaller compared to Unoperated Control animals (LSDs, *ns*). Soma size of RDLN motoneurons in PEG-fused Single Cut or Allograft animals also did not significantly vary by PO survival time (PEG-fused Single Cut [F(1,15) = 0.59, *ns*; PEG-fused Allograft [F(1,23) = 0.77, ns] animals; Fig 4E and 4F).

#### BHRP-labeled somata

In Unoperated Control animals, the mean cross sectional area of TA motoneuron somata was 975.4 ± 42.9 μm^2^ (Table 2), and did not differ from those of L3/L4 motoneurons labeled on the PNI side of Single Cut or Allograft animals with or without subsequent PEG-fusion [F(4,27) = 1.23, *ns*; PEG-fused Single Cuts, 849.2 ± 36.5 μm^2^; PEG-fused Allografts, 834.3 ± 69.0 μm^2^; Negative Control Single Cut, 870.0 ± 106.4 μm^2^; Negative Control Allograft, 799.3 ± 71.2 μm^2^] (Table 2). Soma size of BHRP-labeled L3/L4 motoneurons in PEG-fused Single Cut or Allograft animals also did not vary by PO survival time, with no significant correlation between soma size versus days PO (Single Cut PEG-fused [F(1,11) = 0.16, *ns*] or Allograft PEG-fused [F(1,8) = 3.82, *ns*] animals).

In Unoperated Control animals, the mean cross sectional area of RDLN motoneuron somata was 744.3 ± 39.6 μm^2^ (Table 2), and did not differ from those of L5/L6 motoneurons labeled on the Single cut or Allograft side of axotomized animals with or without subsequent PEG-fusion [F(4,15) = 0.52, *ns*; PEG-fused Single Cuts, 779.3 ± 80.1 μm^2^; PEG-fused Allografts, 787.8 ± 48.5 μm^2^; Negative Control Single Cut, 630.1 ± 28.1 μm^2^; Negative Control Allograft, 908.4 ± 71.2 μm^2^] (Table 2). Soma size of BHRP-labeled L5/L6 motoneurons in PEG-fused animals also did not vary by PO survival times, with no significant correlation between soma size versus days PO in either Single Cut PEG-fused [F(1,5) = 0.67, *ns*] or PEG-fused Allograft [F(1,6) = 0.07, *ns*] animals.

### Retrograde BHRP spinal cord labeling

Injection of BHRP into the TA successfully labeled ipsilateral motoneurons in all treatment groups. A mean of 33.3 ± 4.6 motoneurons per animal was labeled with BHRP, and this did not vary across groups [F(2,31) = 1.09, *ns*].

**Fig 5:**
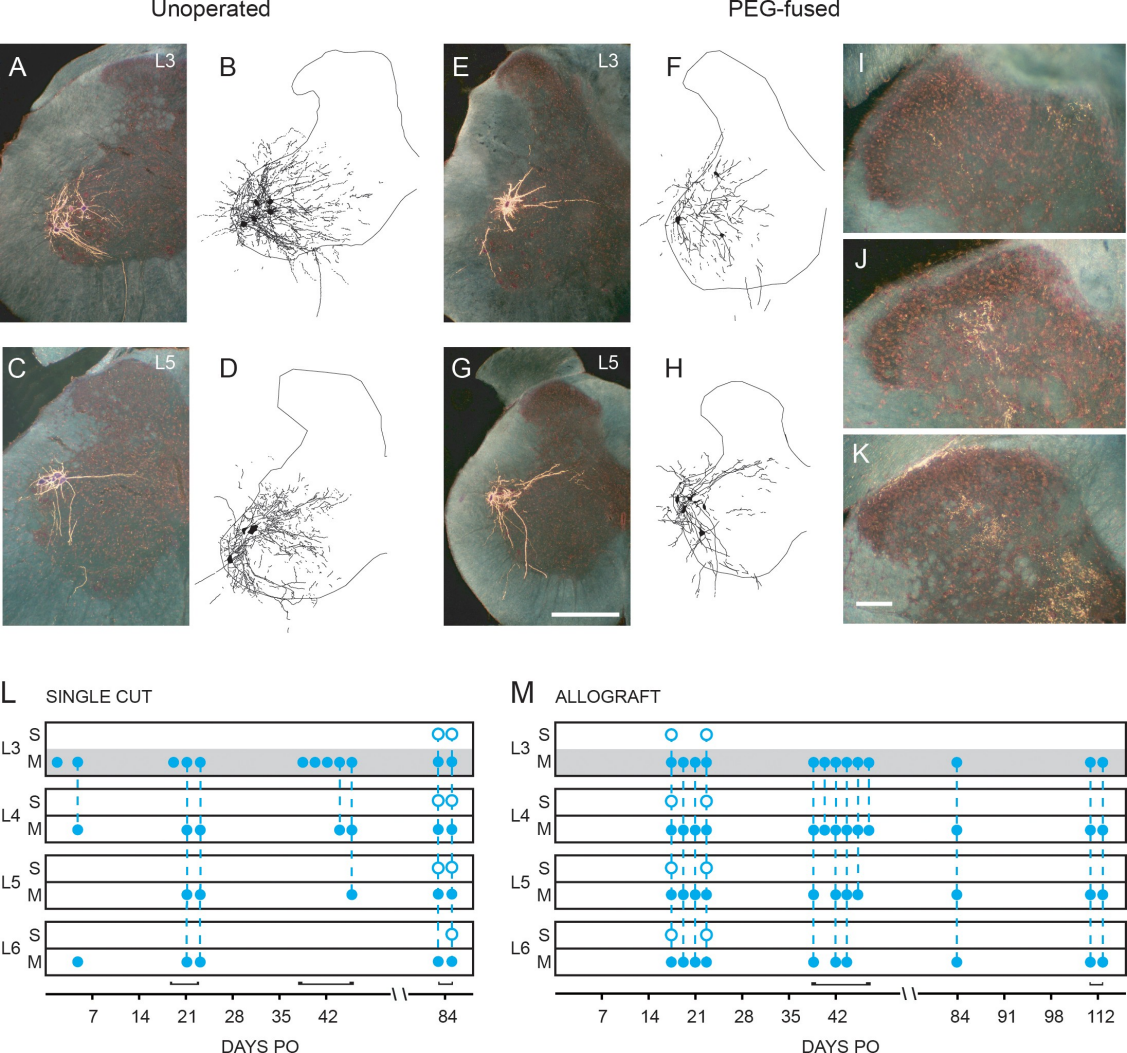
Representative BHRP-labeled motoneurons and primary sensory afferent spinal projections. **(A-H)** Darkfield digital micrographs of transverse hemisections through lumbar spinal cords and computer-generated reconstructions of motoneuron somata and dendritic processes from Unoperated Control animals following injection of BHRP in the TA **(A, B)** or FDB **(C, D)** muscles, and into the TA at 42 days PO of PEG-fused Single Cut **(E, F)** or Allograft **(G, H)** animals. In Unoperated Control animals, BHRP injection into the TA muscle labeled motoneurons in the L3 spinal segment **(A, B)**, while injection into the FDB labeled motoneurons in the L5/L6 spinal segments **(C, D)**. After PEG-fusion repair, BHRP injection into the TA often labels original appropriate motoneurons in L3 segments as well as atypical, inappropriate motoneurons in other spinal segments, e.g., L5 **(G, H)**. Scale bar = 500 μm. **(I-K)** Anomalous sensory afferent terminal labeling in dorsal horn lamina I-V of L3 through L6 segments at 84 days PO in PEG-fused Single Cut animals **(I, J)** and a PEG-fused Allograft animal at 17 days PO **(K)**. Scale bar = 100 μm. **(L, M)** Pattern of motoneuron and sensory labeling in spinal cord segments L3—L6 after injecting BHRP into the TA in PEG-fused Single Cut **(L)** and Allograft **(M)** animals. S: Sensory, M: Motor. Gray L3 M bar indicates normal location of TA motoneurons in Unoperated Controls. Blue open circles indicate location of sensory terminal label; filled circles indicate location of BHRP-labeled motoneurons. Vertical dashed lines represent labeling across multiple lumbar sections within individual animals sampled at a given PO time.

In Unoperated Control animals, following TA injection, labeled motoneurons were observed in their typical, previously reported [36] location in the lateral motor column in the L3 spinal segment (Fig 5A). Dendritic arbors were strictly unilateral, with extensive ramification along the lateral edge of the gray matter and in the lateral funiculus, as well as radially throughout the ventral horn. Following flexor digitorum brevis (FDB) injection, labeled motoneurons were observed in their typical, previously reported [36] location in the RDLN in the L5 and L6 spinal segments (Fig 5C). RDLN dendritic arbors were strictly unilateral, with ramification along the lateral edge of the gray matter and in the lateral funiculus, and with a pronounced medial projection. As expected for our small injection volumes and low concentration of BHRP [33], no sensory afferent terminal labeling occurred in the left or right side of Unoperated Control animals or on the unoperated right side of Negative Control Single Cut or Allograft animals.

In PEG-fused Single Cut animals, retrograde labeling on the unoperated control side animals was consistent with that of Unoperated Control or Single Cut Negative Control animals. On the PEG-fused side of Single Cut animals, retrograde labeling of motoneurons after BHRP injection of the TA muscle was present at the earliest time point (2 days PO), and through the latest time point (84 days PO) examined (n = 13; Fig 5E and 5L). In all but one case, BHRP-labeled motoneurons were present in the L3 spinal segment, indicating re-pairing of proximal motor axons to appropriate distal counterparts. However, in 6/13 individual Single Cut PEG-fused animals, BHRP-labeled motoneurons were observed in anomalous spinal segments, extending caudally into L5/L6, consistent with re-pairing of proximal motor axons to inappropriate distal counterparts (Fig 5L). Anomalous sensory afferent terminal labeling in PEG-fused Single Cut animals was also observed at 84 days in lamina I-V of the dorsal horns of the L3 through L6 segments (Fig 5I and 5J). The distribution of BHRP-labeled sensory terminals varied from being restricted to the medial aspect of the dorsal horn, typical of the termination of sensory fibers from the tibial nerve branch of the sciatic [47], or extending more laterally throughout the entire termination field for sensory fibers from the sciatic nerve [47].

In PEG-fused Allografts as reported above for PEG-fused Single Cuts, retrograde labeling on the unoperated side was consistent with that of Unoperated Control animals. In contrast to Single Cut animals, on the PEG-fused Allograft side, retrograde labeling of motoneurons after BHRP injection of the TA muscle (Fig 5G and 5M) was not observed at 2-3d PO. Rather, at 2–14 PO (n = 16), we failed to find any evidence of BHRP labeling on the PEG-fused Allograft side at any spinal level. Retrograde labeling of motoneurons was not observed in PEG-fused Allografts until 17 days PO, and was present thereafter through the latest time point (112 days PO) examined (n = 13; Fig 5M). In all cases, BHRP-labeled motoneurons were observed in the L3 spinal segment, indicating re-pairing of proximal motor axons to appropriate distal counterparts. However, in all cases, BHRP-labeled motoneurons were almost always (11/13 individual animals) also seen in anomalous spinal segments extending caudally into L5/L6, consistent with re-pairing of proximal motor axons to inappropriate distal counterparts (Fig 5M). Anomalous sensory afferent terminal labeling in PEG-fused Allograft animals was observed only at 17 and 23 days PO, and was present in the dorsal horns of the L3 through L6 segments (Fig 4K). In both of these cases, the distribution of BHRP-labeled sensory terminals extended throughout the entire termination field for sensory fibers from the sciatic nerve [47].

As expected, retrograde labeling of motoneurons was absent on the axotomized side of Negative Control Single cut or Negative Control Allograft animals for at least 21 days PO. Weak labeling of motoneurons in the L3-L6 spinal segments was observed in one Negative Control Single Cut and one Negative Control Allograft animal at 42d PO, with only faintly labeled somata and proximal dendrites. No sensory afferent terminal labeling was present in the nine Negative Control Single Cut or Negative Control Allograft animals.

### Motoneuron dendritic morphology

#### L3/L4 spinal segments

**Fig 6:**
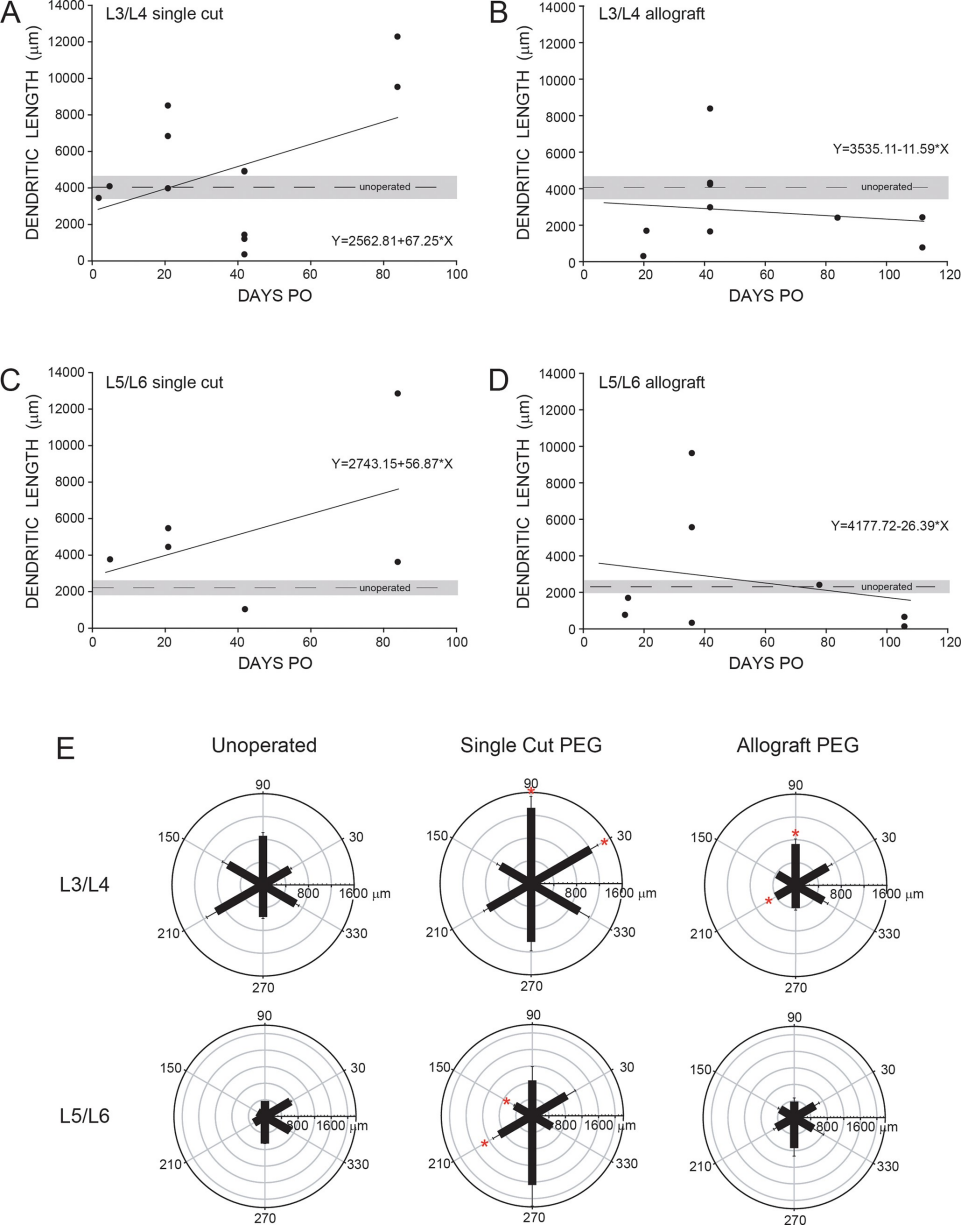
Dendritic length and reorganization. Dendritic lengths of BHRP-labeled motoneurons in the L3/L4 **(A, B)** or L5/L6 **(C, D)** spinal segments of PEG-fused Single Cut and Allograft animals versus PO time. Mean dendritic lengths of TA or FDB motoneurons from unoperated animals shown by dashed lines and ± SEM gray bar; filled circles show individual values for PEG-fused animals. **(E)** Polar plots of total length of dendritic material divided into radial sectors for measure of motoneuron dendritic distribution in 6 bins of 60° each. Bar lengths represent means ± SEM. * indicates significantly different from Unoperated Controls (*p < 0*.*05*).

In Unoperated Control animals, TA motoneuron dendrites had a mean length of 4023.7 ± 604.7 μm (Table 2). On the axotomized side in L3/L4 spinal segments of Single Cut or Allograft animals with PEG-fusion, the mean dendritic lengths of motoneurons labeled after BHRP injection of the TA animals (PEG-fused Single Cuts, 5071.3 ± 1037.5 μm; PEG-fused Allografts, 2887.3 ± 734.8 μm) (Table 2) did not differ from those of Unoperated Control animals [F(2,31) = 1.69, *ns*]. Dendritic lengths of L3/L4 motoneurons in both types of PEG-fused animals did not vary with PO survival times, i.e., no significant correlation between dendritic length versus days PO in either PEG-fused Single Cuts [F(1,11) = 3.17, *ns*] or PEG-fused Allografts [F(1,9) = 0.24, *ns*] animals (Fig 6A and 6B). Although faint labeling of motoneuron somata and proximal dendrites was present in the L3/L4 spinal segments of two Negative Control animals, labeling was insufficient to permit dendritic reconstruction.

Although no significant differences in dendritic length were observed for motoneurons in PEG-fused Single Cut or Allograft rats compared to Unoperated Controls, the distribution of dendrites was altered, resulting in a significant group x location interaction [F(22,341) = 2.21, *p* < 0.002]. In Unoperated Control animals, TA motoneuron dendrites were radially organized around the motoneuron somata (Fig 6E), with the largest concentrations of dendritic material located at 60° to 120° (20.5%) and 180° to 240° (20.4%). In PEG-fused Single Cuts, the distribution of dendrites of L3/L4 motoneurons now projecting to the TA was altered with significant increases in dendritic material at 0° to 60° and 60° to 120° [Fs(1,22) > 4.97, *p*s < 0.04]. Dendritic distributions were also altered in PEG-fused Allografts, with significant decreases in dendritic material at 60° to 120° and 180° to 20° [Fs(1,20) > 4.49, *p*s < 0.04].

#### L5/L6 spinal segments

In Unoperated Control animals, RDLN motoneuron dendrites had a mean length of 2265.4 ± 336.6 μm (Table 2). Mean dendritic lengths of motoneurons on the axotomized side in the L5/L6 spinal segments labeled after BHRP injection of PEG-fused Single Cut or Allograft animals (PEG-fused Single Cuts, 5136.4 ± 1642.8 μm; Allograft PEG-fusion repair, 2615.2 ± 1175.9 μm) (Table 2) did not differ significantly from those of Unoperated Control animals [F(2,18) = 1.73, *ns*]. Dendritic lengths of L5/L6 motoneurons in PEG-fused Single Cut or Allograft animals did not vary with PO survival time, i.e., no significant correlation between dendritic length versus days PO in PEG-fused Single Cut [F(1,5) = 1.15, *ns*] or PEG-fused Allograft [F(1,7) = 0.60, *ns*] animals (Fig 6C and 6D). Although faint labeling of motoneuron somata and proximal dendrites was present in the L5/L6 spinal segments of two Negative Control animals, labeling was insufficient to permit dendritic reconstruction.

Although no significant differences in dendritic length were observed for motoneurons in PEG-fused Single cut or Allograft animals, the distribution of dendrites was significantly altered, resulting in a significant group x location interaction [F(22,198) = 1.60, *p* < 0.05]. In Unoperated Control animals, RDLN motoneuron dendrites were asymmetrically organized around the motoneuron somata (Fig 6E), with the largest concentrations of dendritic material located at 60° to 120° (24.8%) and 300° to 360° (25.0%). In PEG-fused Single Cut animals, the distribution of dendrites of L5/L6 motoneurons now projecting to the TA was altered, with significant increases in dendritic material at 120° to 180° and 180° to 240° [Fs(1,11) > 5.58, *p*s < 0.04]. Dendritic distributions were not significantly altered from those of Unoperated Control animals compared to PEG-fused Allograft animals [F(11,143) = 0.67, *ns*].

## Discussion

Consistent with our previous reports [25, 26], animals with PEG-fusion repair of single cut or PNI ablation sciatic nerve lesions rapidly (within seconds to minutes) re-established axolemmal and axoplasmic continuity. Immediate restoration of axonal continuity has been assessed in this and/or previous reports by conduction of CAPs across the PEG-fused lesion sites [23–27], and by CMAPs stimulated proximal to lesion sites and recorded from muscle groups distal to all repair sites [25, 26]. PEG-fusion also reduced or prevented Wallerian degeneration of axons in nerve segments distal to the lesion sites, reduced muscular atrophy, and maintained innervation of NMJs. Voluntary behaviors were restored 2–6 weeks after PEG-fusion repair of single cut or ablation PNIs. Our current results confirm prior reports that PEG-fusion connects axons after severance, and we now describe for the first time the extent of proximal-distal axonal mispairings and its effects on spinal remodeling.

Data presented herein confirmed that PEG-fusion did *not* specifically reconnect the severed original distal and proximal stumps of motor and sensory axons, but rather non-specifically connected closely apposed axons of any type at sites of PEG-fusion, resulting in inappropriate motor to motor, or even motor to sensory connections. Although the number and somal size of motoneurons was unaffected, dendritic distributions of motoneurons were altered, indicating that PEG-fusion preserves spinal motoneurons, potentially reorganizing their connectivity. Incorrect target projections by motoneurons after axotomy with PEG-repair also did not result in motoneuron death at any PO time. This result is important, as induced death of motoneurons results in morphological changes in surviving motoneurons [37]; thus, the dendritic changes we observed in PEG-fused animals (see below) cannot be ascribed to the death of neighboring motoneurons.

In brief, PEG-fusion immediately preserves spinal motoneurons, changes their peripheral connectivity, and alters dendritic organization. This spinal reorganization may contribute to the remarkable behavioral recovery that is not present at the time of axonal repair, but develops in the weeks after PEG-repair. These longer term CNS (and PNS) plasticities are discussed in the following sections.

### Spinal cord labeling

Retrograde labeling of spinal motoneurons was restored in 0–2 days after Single cut PEG repair and 17 days after Allograft PEG repair. This difference between the two PEG repair types in the time required to re-establish active retrograde axonal transport may reflect a difference in injury (one fused cut vs. two), differences in cytoskeletal dynamics to repair damaged microtubule tracks, defects in motor proteins, or some combination thereof. More importantly, this retrograde labeling via distal axons maintained by PEG-fusion is present far sooner than could be accomplished by reinnervation by regenerating axons. That is, in Negative Control Single Cut animals, reinnervation of NMJs by regenerating axons was not robust even at 42d PO, and then highly variable (see Table 1). This partial reinnervation by regenerating axons resulted in concomitant weak labeling of motoneurons in the L3-L6 spinal segments, present in only two of the six Negative Control Single Cut animals examined at 42 days PO. Because some reinnervation by regenerating axons occurs by 42 PO, spinal labeling observed in PEG-fused animals at this and later time points could reflect PEG fused altered connections (mispairings) and/or non-specific outgrowth of regenerating axons.

After both types of PNIs followed by PEG repair, BHRP-labeled motoneurons were observed in the appropriate spinal cord segment (i.e., L3 after injection into the TA). BHRP-labeled motoneurons were also observed in inappropriate spinal segments (e.g., L5 and L6, whose motoneurons typically innervate intrinsic muscles of the foot), but the incidence of inappropriate labeling differed between PEG-fusion repair of single cut versus ablation type PNIs. In PEG-fused Single Cut animals, fewer than half (46%) of the animals had BHRP-labeled motoneurons outside of the L3 spinal segment following injection into the TA muscle. In contrast, all of the PEG-fused Allograft animals also had BHRP-labeled motoneurons in inappropriate spinal segments. Because these patterns of labeling were present in both groups prior to what could result from reinnervation by non-specific outgrowth of regenerating axons, they must result from PEG-fusion. The differences in the degree of specificity across the two repair types might in part result from the organization of the axons within rat sciatic nerves. In rat sciatic nerves, axons may be organized topographically, and motor axons innervating particular muscles or sensory axons from specific cutaneous areas course together in increasingly distinct fascicles in more distal portions of the nerve [48]. In PEG-fused Single Cut nerves, realignment of appropriate fascicles in the proximal and distal nerve segments might occur and thereby account in part for the reduced incidence of inappropriate motoneuron labeling. In contrast, the exact number of sciatic axons and their specific arrangement may slightly differ in the right/left peripheral nerves of a given rat, much less between different individual rats [49]. Additionally, donor nerve allografts are typically sized to be larger than the ablated section in the host nerve, and alterations in fascicular position within the graft over long distances would contribute to misalignment. This misalignment, combined with the potentially different axonal numbers and fascicular organization within the graft, would necessarily produce significantly more mis-alignment and subsequently, extensively altered labeling of motoneurons in PEG-fused Allograft nerves.

Sensory afferent terminal labeling in the spinal cord was never observed in Unoperated Control, Negative Control Single Cut or Negative Control Allograft animals, confirming that BHRP uptake was restricted to motor axons that have the GM1 ganglioside that produces transmembrane transport of BHRP [34]. In contrast, labeling of sensory afferent terminals was observed in PEG-fused animals, consistent with the possibility that distal motor axons could be PEG-fused with proximal sensory axons in single cut or ablation-type PNIs. Sensory labeling was present at 84 days PO in PEG-fused Single Cut animals. Although this is a sufficient period of time to allow reinnervation by regenerating axons, given that BHRP at the concentration and volume we used is not taken up by peripheral sensory axons, this almost-certainly reflects a pairing of proximal sensory axons to distal motor axons. Similarly, sensory labeling was present in two PEG-fused Allograft animals at 17 and 23 days PO respectively. This labeling was present far sooner than could be accomplished by reinnervation by regenerating axons, and is consistent with PEG-fusion non-specifically joining motor and sensory proximal/distal cut ends.

Motoneuron pools are organized in tightly regulated arrangements, and high reinnervation specificity is often assumed necessary for behavioral recovery after PNIs. Incorrect reinnervation of musculature after PNI is often assumed to account for poor functional and voluntary behavioral outcomes due to disruption of coordinated activation [19, 20, 50]. However, this may hold only for regeneration after nerve crush injuries of relatively short length in which intact endoneurial tubes connect proximal distal axons. Single-cut sciatic nerves repaired with neurorrhaphy regenerate motor axons with 40–70% specificity, while PNIs that are microsutured with precise individual fascicular repair after single cuts show greater specificity of regeneration (60–90%), but no significant recovery of voluntary behaviors compared to standard neurorrhaphy [51]. Nerve transfers, in which nearby healthy donor nerves are sutured to the distal site of an injured nerve, connect motoneurons to inappropriate muscles, but often produce excellent behavioral outcomes. By increasing the speed of target reinnervation, nerve transfers may induce CNS or PNS plasticites and relearning for functional and voluntary behavioral recovery over time [52]. Post-natal behavioral recovery in response to PNS mis-wirings can also occur by environmental enrichment [50]. Extreme levels of CNS rearrangements have been studied in rotations of sensory connections from the back and belly skin in frogs to produce changes in voluntary behaviors [53]. All these data suggest that CNS plasticities can play a role in pattern relearning for motoneurons that survive PNIs. These examples provide evidence of alternate pathways for restoration of function independent of reinnervation specificity. Alterations of spinal and supra-spinal pathways provide a potential mechanism for such phenomena.

### Motoneuron dendritic morphology

Spinal motoneurons have extensive dendritic arbors that span several spinal segments and account for up to 97% the surface area of a neuron [54] with 20,000–50,000 synaptic inputs [55]. Most motoneuron synapses are made on dendrites [56] and differences in dendritic branching patterns, distribution, and overall shape determine important functional properties (e.g., [57–59]). Further, motoneurons innervating different muscles are associated with distinct dendritic morphologies [60, 61], as we observe in the present study.

Dendritic lengths did not significantly differ between PEG-fused motoneurons and their counterparts in Unoperated controls. This preservation of dendritic length by PEG fusion contrasts with atrophic changes typically observed after axotomy [11–13] that can be reversed upon muscle reinnervation by outgrowth [12, 14, 15]. Although dendritic length was unaffected, dendritic distributions of motoneurons projecting to the TA muscle in PEG-fused Single Cut or Allograft animals were significantly altered compared to those of Unoperated controls. Given that motoneurons innervating different muscles have distinct dendritic morphologies, this mis-pairing of motoneurons to the TA muscle could potentially reflect this reassignment. However, BHRP-labeled motoneurons in the appropriate spinal cord segment (L3) also had dendritic distributions that differed from those of unoperated animals. This reorganization of motoneuron dendrites could result from axotomy induced deafferentation and dendritic retraction [62], a failure of PEG fusion to repair sensory afferents, and/or sensory misconnections induced by PEG-fusion repair. In addition, changes in descending information from propriospinal tracts or supraspinal projections to compensate for mispairings might also produce dendritic reorganization. Alterations in dendritic distributions might also differ after single cut or allograft PEG repairs. However, it is important to note that part of the changes in dendritic distribution we observed after PEG fusion in each animal are specific to that animal, reflecting the exact nature of the injury, surgical repair, and central plasticity unique to that animal. That is, some aspects of the dendritic response are common to all animals and some are unique to each animal.

While the focus of this study was to examine the changes in motoneuron location and morphology, other mechanisms of CNS plasticity might also play a role in PEG-fusion restoration of voluntary behaviors otherwise lost after PNIs. For example, regulation of motoneuron gene expression, excitability, remodeling of the number and types of synaptic inputs, and supraspinal contributions to CNS plasticities might contribute to the re-wiring of CNS or PNS connection of PEG-fused proximal-distal axons. In addition to such CNS plasticities, PNS plasticities might produce changes in muscle fiber type or synapse remodeling. Such changes in the CNS and PNS will need to be considered together to provide a better understanding of the intricate communication and synergy between these systems to achieve functional reorganizations in response to PNIs.

## Conclusion

PEG-fusion repair produces dramatic functional improvement despite alterations in re-innervation specificity, which implies that altered, immediate reconnections induced by PEG-fusion might be far superior to specific reconnections made by regenerating axons requiring protracted periods to reach distal targets. The dramatic functional improvement we observed for PEG-fused animals despite the altered connections of motoneurons to distal axons could be due in part to CNS plasticities reflected in dendritic reorganizations, potentially providing a substrate for changes in afferent input. A better understanding of the phenomena responsible for the restoration of voluntary behaviors after PEG-fusion repair might help guide clinical interventions to different types of PNIs, as well as efforts to increase specific CNS/PNS plasticities to further increase recovery. PEG-fusion repair and subsequent rehabilitative efforts aimed at optimizing immediate altered connections might provide a better approach than current procedures to repair single cut or ablation-type PNIs.

## Abbreviations

BHRP: cholera toxin B conjugated horseradish peroxidase
CAP: compound action potential
CMAP: compound muscle action potential
CNS: central nervous system
NMJ: neuromuscular junction
PEG: polyethylene glycol
PNI: peripheral nerve injury
PNS: peripheral nervous system
PO: post-operative
SFI: sciatic functional index
TA: tibialis anterior
RDLN: retrodorsolateral nucleus
